# A fine line between obsessive–compulsive disorder (OCD) and psychotic disorder: a case report

**DOI:** 10.1186/s13256-026-06404-1

**Published:** 2026-07-24

**Authors:** Natalia Chechko, Stefanie Weisenfeld, Susanne Nehls

**Affiliations:** Department of Psychiatry, Psychotherapy and Psychosomatics, Faculty of Medicine, University Hospital RWTH Aachen, Pauwelsstraße 30, 52074 Aachen, Germany

**Keywords:** Obsessive compulsive disorder, Psychosis, Misdiagnosis

## Abstract

**Background:**

Differentiating obsessive–compulsive disorder (OCD) from psychotic disorders can be problematic, especially when OCD symptoms present with poor insight and psychotic-like intensity.

**Case presentation:**

This case report describes a 44-year-old white male, whose symptoms began in his late teens. Clinical documentation, however, began in 2009, when he was first hospitalized. Although he was initially diagnosed with psychosis, his intrusive quasi-delusional fears and compulsions, accompanied by experiences of being watched and abstract ideas about the “ego” with limited insight, led to diagnostic uncertainty. While antipsychotic treatment was ineffective, the initiation of paroxetine in 2011 resulted in substantial improvement, suggesting an OCD etiology. Despite later revision of his diagnosis (after more than 10 years following manifestation) toward an OCD spectrum disorder, the patient continued to believe he had psychosis and developed obsessive fears of becoming psychotic, likely influenced by prior clinical labeling.

**Conclusion:**

This case underscores the importance of careful differential diagnosis in OCD with poor insight. Early recognition of OCD can significantly alter prognosis and treatment course, avoiding prolonged exposures to neuroleptics.

## Introduction

Both psychosis and obsessive–compulsive disorder (OCD) are characterized by disordered thought content. In OCD, repetitive thoughts are intrusive, inappropriate, and distressing, whereas in psychosis, thoughts are typically fixed delusions, and false beliefs that persist despite evidence to the contrary. Most people with OCD know or suspect that their obsessive thoughts are not true, but they have difficulty nonetheless to disengage from those thoughts or stop their compulsive behaviors. In contrast, those experiencing psychosis often exhibit an impaired understanding of their condition, firmly believing in the validity of their delusional convictions [1, 2].

According to the DSM-5 diagnostic criteria, OCD patients may also lack insight into their condition, broadening the scope of OCD diagnosis. OCD patients with poor insight tend to believe that their beliefs are either likely or certainly true [3]. Approximately 13–36% of OCD patients lack insight [4], exacerbating the disease severity and complicating treatment and the social burden.

This lack of insight poses a formidable diagnostic difficulty, particularly when obsessive thoughts cannot be clearly distinguished from delusional beliefs. The diagnostic process becomes especially challenging when the boundary between obsession and delusion is blurred, resulting in frequent misdiagnoses. Interpreting a compulsive disorder as a psychotic disorder can have profound consequences for long-term outcomes and prognosis. The following case report, resulting from an extended period of observation, illustrates how OCD with poor insight, when initially misdiagnosed as psychosis, can significantly influence the course of illness, underscoring the critical importance of accurate diagnosis.

## Case presentation

This case report involves a 44-year-old white male patient (*nomen nescio*, hereafter called NN). The first inpatient presentation of NN was in 2009 due to his experience of diffuse anxieties. Bus or train rides and the use of elevators were proving particularly problematic for him. He would also not dare to leave the house because of his fear of hitting others. The patient was already placed on clomipramine and quetiapine as an outpatient. As the symptoms persisted, alongside a delusional experience involving the feeling of being observed and the conviction that he could read the genes of other people, inpatient admission was initiated with the suspicion of psychotic experience. With this, a prolonged and difficult treatment course began, during which the patient remained clinically unstable for at least two years.

In the subsequent therapeutic course, the dose of quetiapine was gradually increased to 800 mg per day, without meaningful improvement. A switch to risperidone, at 6 mg per day, led to the side effect of motor restlessness. Consequently, clozapine was introduced. Despite these changes, NN remained clinically unstable for an extended period, requiring hospitalization for the majority of the time. A close observation of the patient revealed that he spent a lot of time washing his hands. It also became apparent that NN was not, in fact, scared of the car ride itself, but rather suffered from intrusive fears of losing control, specifically that he might open the car door while the vehicle was in motion. Similarly, his avoidance of higher floors was driven by fears that he might impulsively jump out of a window. Due to the doctors' renewed suspicion that he might harbor obsessive thoughts (in addition to paranoia), 15 mg of escitalopram was administered daily, in addition to 300 mg of clozapine. This, however, did not yield long-term stability, with the thought of being on his own again causing NN great duress.

As part of the somatic evaluation, NN was diagnosed with multiple sclerosis, which led to the assumption of an organically caused mental disorder (organic schizophreniform disorder). The patient rejected any treatment of this disease, forcing the doctors to focus only on psychiatric therapy**.** In the meantime, the patient’s symptomatology became increasingly complex, further complicating the diagnostic process. His own accounts of obsessive thoughts concerning the location of the “ego”, together with experiences of depersonalization, prompted the continuation of antipsychotic therapy, as these phenomena were interpreted as psychotic. The interpretation was based on the abstract nature of the thoughts and the patient’s limited capacity for critical appraisal. Retrospectively, however, it became evident that these experiences were consistent with obsessive–compulsive pathology. While his thoughts were intrusive and anxiety-provoking, he did not experience a loss of reality or a true break from reality, which would be characteristic of psychotic symptoms. At the same time, his obsessions, particularly his fear of accidentally harming himself, and the associated ritualized compulsive behaviors became progressively more prominent. Finally, an outpatient switch to paroxetine 20 mg per day in 2011 resulted in a significant reduction of symptoms for several years. However, psychosis remained the discharge diagnosis. In summary, between 2009 and 2011, the patient experienced near-continuous instability, requiring prolonged hospitalization and treatment with three different antipsychotics, none of which led to meaningful clinical improvement. While prominent compulsive tendencies were recognized, the patient's thoughts were interpreted by the treating physicians as being largely psychotic, rather than obsessive–compulsive, primarily due to their intensity, quasi-delusional quality, and markedly reduced insight. This led to a working diagnosis of psychosis (DD organic schizophreniform disorder), despite ongoing features suggestive of obsessive–compulsive presentation.

In 2017, NN's father died of a brain tumor, which likely triggered a deterioration of the patient’s condition. Once again, he was unable to climb stairs or use an elevator. He felt a strong sense of restlessness and tension, fearing that something bad was going to happen, that he might, for instance, fall over and hurt himself. He also reported that he could not eat anything for fear of choking, thus opting only for soups. He could no longer tolerate being alone due to his obsessive fears of uncontrolled actions. This restricted his lifestyle in such a way that he would not leave his ground-floor apartment for up to one year, forcing his brother to move in with him.

Over time, the OCD progressively worsened as NN increasingly perceived the obsessive thoughts to be ego-syntonic, and lost the ability to understand the connection between his obsessive thoughts and the accompanying fears. As his use of paroxetine led to the development of sexual dysfunction, he refused further treatment with this medication. Subsequently, a variety of antidepressants were started without success and were discontinued (see Table 1). Since the patient was convinced that clozapine would reduce his anxiety and that he was suffering from psychotic experiences, he could not be dissuaded from taking the medication, which was continued at the lowest dosage accepted by the patient (75 mg). After some time, NN tolerated fluvoxamine 150 mg, which led to a stabilization that lasted for three years, during which period no hospital stays were necessary. Clozapine was finally discontinued. Based on the patient’s positive response to paroxetine and fluvoxamine, and the absence of schizophreniform symptoms, the diagnosis of organic schizophreniform disorder was replaced with that of an OCD (DD organic OCD).

**Table 1:** Longitudinal clinical course of patient NN illustrating symptom evolution, diagnostic shifts, and major pharmacological interventions over a 20-year period

Year	Key symptoms / events	Diagnosis / interpretation	Treatment	Outcome
≤ 2008	Long-standing anxiety and obsessive fears	–		
2009	Anxiety, avoidance, delusional ideas	Suspected psychotic disorder	Clomipramine 75 mg/day, Quetiapine 300 mg/day ↑ 800 mg/day	No improvement
2009	Motor restlessness	–	Risperidone 6mg/day	Limited benefit
2009–11	Compulsions; clarified obsessions Somatic workup Severe avoidance; fear of being alone	Obsessive-compulsive disorder features noted Encephalomyelitis disseminata → presumed organic psychosis Psychotic interpretation maintained	Psychotherapy + exposure Somatic tx refused Clozapine 300 mg /day + escitalopram 15 mg/day	Minimal effect No stability
2011	Persistent obsessions, depersonalization	Psychosis diagnosis retained	Paroxetine 20 mg	6 years stability
2011–16	Stable	Psychosis (discharge dx)		Functional stability
2017	Relapse after father’s death; severe avoidance; choking fears	Diagnostic reconsideration to obsessive-compulsive disorder / affective delusional disorder		Major deterioration
2018–19	Ego-syntonic obsessions Patient’s conviction of psychosis	Obsessive-compulsive disorder predominant	Paroxetine stopped (sexual SEs) Clozapine 137.5 mg/day Aripiprazole 10 mg/day Mirtazapin 15 mg/day	Worsening
2019–22	Stable period	Obsessive-compulsive disorder	Fluvoxamine 150 mg	3 years stability
2022–23	Frequent admissions; reassurance seeking; somatization	Chronic obsessive-compulsive disorder	Escitalopram 5 mg/day Cariprazine 1,5 mg/day Pregabalin 25 mg/day Valdoxan 25 mg/day Clozapine 75 mg/day	Each refused after several weeks
2024	Persistent obsessive-compulsive disorder	Obsessive-compulsive disorder	Clozapine 75 mg/day	Assisted living

Since 2022, however, inpatient admissions have increased again due to the recurrence of previous issues, coinciding with the discontinuation of fluvoxamine. The patient refused fluvoxamine for the same reasons as paroxetine. Instead, the patient and his parents insisted on restarting clozapine, which was reintroduced at a low dose due to its calming effect. Because the multiple sclerosis symptoms remained in remission without treatment, and there was no morphological evidence of progression of the disease over a period of 13 years, an organic cause was also deemed unlikely. In sum, more the 10 years after the first hospitalization, the diagnosis of OCD was established, and that of psychosis or organic origin was eliminated as an incorrect assessment. Ironically, while the doctors became convinced of the OCD diagnosis, the patient’s obsessive thoughts had led him to believe that he was suffering from psychosis. With most of his obsessive thoughts since 2022 involving the fear that something bad can happen to him, he feels relieved to be in the hospital, where doctors are available around the clock. An overview of the longitudinal clinical course and major pharmacological interventions is shown in Table 1.

His current psychopathology is marked by pronounced reassurance-seeking tendencies, as he attempts to prolong hospital stays and vehemently demands somatic investigations. The patient has also developed somatization tendencies, reporting multiple physical complaints such as “brain pain”, headaches, “eye pain”, and a sensation of dizziness and fainting. According to him, all of these symptoms have been caused by selective serotonin reuptake inhibitors (SSRI) medication and exposure training. He rejects the notion that his physical complaints and feelings of dizziness are expressions of his fears and obsessions, insisting instead on intensive somatic evaluation, despite repeated unremarkable findings. With his OCD now persisting for about 20 years, NN exhibits strong illness-maintaining behavior despite a lack of resources. In spite of intensive psychoeducation, he cannot accept a diagnosis of pure OCD and repeatedly refuses appropriate therapy. To make matters worse, the illness-supporting behavior of his mother and brother (who are convinced that he is psychotic and prevent the discontinuation of clozapine) limits his treatment options and negatively impacts his therapy adherence.

The patient’s addiction history is unremarkable apart from a single consumption of cannabis at the age of 16. He has three siblings, and with the exception of one brother who also suffers from OCD (his other siblings, two sisters, are healthy), there is no family history of psychiatric illnesses. NN graduated from high school at the age of 19, but did not undergo any training and did not work. He has no social contacts apart from his family. His father was a doctor, and his mother a housewife. The family is emotionally distant, despite being financially stable and well-situated.

The psychological test in 2022 yielded unremarkable results: average verbal intellectual performance (WST) and processing speed (TMT-A) were strong, selective attention (d2-R) intact, executive functions were strong in cognitive flexibility (TMT-B) and in the area of divergent problem-solving ability (RWT) mostly impaired, short-term and working memory (WMS-R) were intact, logical memory (WMS-IV) was impaired, mild depressive symptoms (BDI-II).

This case report illustrates the gradual evolution in the diagnosis of a 44-year-old male patient with complex psychiatric symptoms. In 2009, the patient was first admitted due to diffuse anxieties and delusional experiences, including feeling observed and believing he could read others' genes. With the initial diagnosis being psychosis, he was treated with multiple antipsychotics, including quetiapine, risperidone, and clozapine, but without significant improvement. Over time, signs of obsessive–compulsive tendencies, such as intrusive thoughts and fear of losing control, became more evident. In 2011, he was switched to paroxetine 20 mg, which led to a significant reduction in symptoms, though the psychotic diagnosis remained unchanged. In 2017, the death of his father triggered a worsening of his symptoms, with an increase in OCD behaviors, including a fear of choking and avoiding being alone. He refused further treatment with paroxetine due to sexual dysfunction, but tolerated fluvoxamine 150 mg, which led to stabilization. By 2022, inpatient admissions increased following the discontinuation of fluvoxamine. Finally, after more than a decade, the diagnosis of OCD was confirmed, and the earlier psychotic diagnosis was ruled out. The symptoms of OCD persist, with strong illness-maintaining behaviors. Family support continues to hinder treatment adherence, as the patient remains convinced of his psychotic state, leading to ongoing treatment with a low dose of clozapine.

## Discussion

The patient was initially misdiagnosed with psychosis due to his lack of insight into the irrational nature of his intrusive thoughts. Despite suspecting OCD, the doctors were swayed by the intensity and delusional quality of his thoughts, which led to the inaccurate diagnosis and prolonged periods of incorrect treatment. This raises the question as to whether a correct diagnosis could have been made earlier. Throughout the course of the illness, the patient's cognitive performance has remained consistently intact. While mild cognitive impairments can occur in OCD, and cognitive decline is not universal in schizophrenia-spectrum disorders, progressive and functionally disabling cognitive deterioration is more characteristic of chronic psychotic illness than of OCD, particularly when negative symptoms are absent [5]. Another point to consider here is that our patient has remained consistently organized in psychopathological terms, in contrast to psychotic patients who often display incoherence, disorganization, and tangential speech [5]. Finally, the presence of intrusive compulsive urges, a characteristic feature, further supports the diagnosis of OCD. Additionally, the favorable response to the antidepressants, coupled with a lack of improvement under antipsychotic treatment (for example, clozapine), further corroborates the diagnosis of OCD. A sustained improvement and long-term remission were observed following the administration of serotonergic medication, particularly paroxetine.

In many cases, it is difficult to differentiate between psychotic experiences and compulsions because the transition is fluid [6]. For instance, the patient's thoughts about the location of the “Ego” and depersonalization, initially interpreted as psychotic, were later reinterpreted as existential or philosophical obsessions, a subtype of OCD in which individuals become preoccupied with questions related to identity, reality, and the self. Given the nature of these thoughts, they can sometimes be difficult for doctors to interpret as obsessive, thus leading to a misinterpretation, as was the case with our patient.

In addition, obsessions and compulsions can co-occur with psychosis or schizophrenia [7]. Up to 10.6–14% of patients with a first episode of psychosis have been found to have also had an OCD [7, 8]. About 30% of patients suffering from schizophrenia report obsessive–compulsive symptoms [9, 10]. Together, these illnesses can involve ideas, thoughts and impulses to act, which are difficult to understand and seem strange [6]. In OCD, patients tend to have a general insight into the senselessness of their thoughts, which is usually unavailable to patients with psychotic experiences [1, 6, 11]. According to International Classification of Diseases (ICD-10), insight must always be present, whereas according to Diagnostic and Statistical Manual of Mental Disorders (DSM-V), insight must have existed at least at some point in medical history. The problem, however, is that even in OCD, this ability to have insight can fluctuate or at some point be completely absent [6, 11], particularly with long illness duration and severe symptoms. In clinical practice, up to 6% of OCD patients appear to lose insight [8].

The contents of the obsessive thoughts and actions also tend to vary. In OCD, the thoughts are primarily concerning infection, poisoning, pollution and illness, about 50% of obsessions being fears of contamination [12]. Somatizing his fears over time, NN developed headaches and heart pain, as well as increasing dizziness and the feeling of fainting in states of increased tension. After contamination fears, somatic obsessions (33%) are the most common form of obsession [12]. In addition, in the new classification according to ICD-11 and DSM-5, obsessive–compulsive disorders are grouped with somatoform disorders, illustrating the close connection between the two disorders. Tonna et al. [13] have shown that compulsive behaviors are similar in patients with OCD compared to schizophrenic patients, with OCD patients displaying more aggressive, contamination and somatic obsessions than their counterparts with schizophrenia. Also, the belief that thoughts are an input from the outside, with individuals reporting acoustic hallucinations in the form of hearing voices, is an indication of schizophrenic illness [11], which was always missing in our patient.

In our case, it is unclear as to the extent to which clozapine might have led to an exacerbation and chronification of OCD as described in some studies [14]. It has been proved that other antipsychotics such as haloperidol, risperidone and aripiprazole can be part of a therapeutic strategy for refractory compulsion as an augmentation for SSRI [14–16]. The distinction is due, most likely, to the differences in the receptor binding profile of antipsychotics [14]. However, in our patient’s case, the effects of clozapine are unlikely as the symptoms were already present and no worsening of the same was seen following its initiation.

In sum, the key issue in this case was the prolonged misdiagnosis of the patient, which led to several years of incorrect treatment. Initially, there was suspicion of OCD, but due to the intensity and delusional quality of his intrusive thoughts, the diagnosis shifted toward psychosis, which was further reinforced by the patient’s lack of insight into the irrational nature of his thoughts, and his own belief alongside that of his family that he was suffering from psychosis. However, it is crucial to note that this belief eventually became a focal point of the patient's symptomatology. As the treatment continued, the patient’s obsession with the idea of becoming psychotic intensified. Rather than improving in response to appropriate treatments, the misdiagnosis inadvertently led to the formation of a new obsession: the obsessive fear of psychosis. This shift in the patient's cognitive framework, that is, the fear of developing psychosis, eventually became a defining feature of his OCD, further complicating his treatment and creating a feedback loop where his obsession with psychosis perpetuated the belief, despite the lack of core psychotic symptoms.

## Limitation

A key limitation of this case report is its retrospective nature, particularly with respect to certain clinical decisions and diagnostic shifts. The evolving understanding of the patient's symptoms, such as the initial misdiagnosis (psychosis) and subsequent recognition of OCD, is based on a post hoc analysis. Nonetheless, this case highlights the challenges of diagnosing complex, long-standing psychiatric disorders and underscores the need for ongoing clinical reassessment.

## Conclusion

This case emphasizes the critical need for accurate and timely differential diagnosis in complex psychiatric cases, particularly when patients present with multifaceted and long-standing symptoms. The primary learning point is the importance of distinguishing OCD from psychosis. The misdiagnosis of OCD as psychosis, in this case, not only led to inappropriate treatment but also exacerbated the patient's symptoms. Specifically, the prolonged use of clozapine, without a clear indication, posed risks, including metabolic side effects and increased mortality.

The clinicians treating NN ought to have been attentive to the following diagnostic clues. First, the content and the response to intrusive thoughts, such as concerns about the "location of the Ego" and depersonalization, suggest OCD rather than psychosis. Second, the patient's family history of OCD, particularly his brother’s condition, is an important clue. Third, the patient’s response to medications, improvement with paroxetine and lack of response to antipsychotics, should have raised doubts about the original diagnosis. Finally, the persistent obsession with becoming psychotic, despite antipsychotic treatment, suggested that the psychotic diagnosis was not addressing the core symptoms, which were rooted in OCD.

This case also underscores the need for further research into diagnostic tools that can better differentiate between OCD and psychosis, particularly in patients lacking insight into their symptoms. A clearer understanding of these distinctions would improve clinical outcomes and help avoid unnecessary treatments.

## Data Availability

Data sharing is not applicable to this article as no datasets were generated or analyzed during the study. Identifying/confidential patient data cannot be shared.

## Abbreviations

BDI-II: Beck’s Depression Inventory II
OCD: Obsessive compulsive disorder
RWT: Regensburger Wortflüssigkeit [word fluency test]
TMT: Trail making test
WMS-R: Wechsler memory scale revised
WST: Wortschatztest [vocabulary test for verbal intelligence
